# Evaluation of the Nano Liquid Chromatography Coupled With Zeno TOF 7600 Mass Spectrometer for Cell Type-Resolved Spatial Proteomics

**DOI:** 10.1016/j.mcpro.2026.101599

**Published:** 2026-10-13

**Authors:** Keren Zhang, Xi Wang, Wan He, Jiani Wang, An He, Binbin Zheng, Yangqiu Li, Ruilian Xu, Weina Gao, Henry Lam, Ruijun Tian

**Affiliations:** 1 Department of Oncology, The Second Clinical Medical College, The First Affiliated Hospital, Jinan University (Shenzhen People’s Hospital, the First Affiliated Hospital of Southern University of Science and Technology), Shenzhen, China; 2 Shenzhen Key Laboratory of Functional Proteomics, Research Center for Chemical Biology and Omics Analysis, Guangming Advanced Research Institute and Department of Chemistry, School of Science, Southern University of Science and Technology, Shenzhen, China; 3 Department of Chemical and Biological Engineering, The Hong Kong University of Science and Technology, Clear Water Bay, Hong Kong, China; 4 Shenzhen BayOmics Biotechnology Co., Ltd., Shenzhen, China; 5 Guizhou Green Industry Technology Institute, Guiyang, China

**Keywords:** Zeno TOF 7600, nano-LC, DIA, targeted proteomics, spatial proteomics, gastric cancer

## Abstract

In this study, we systematically evaluated the nanoLC-Zeno TOF platform using data-independent acquisition (DIA) and identified 3142 to 7059 protein groups from 1 to 200 ng of standard K562 digest. Additionally, a targeted acquisition strategy detected 184 peptides with enhanced signals using Zeno activation. To achieve cell type-resolved spatial proteomics, we developed a workflow integrating image-guided laser capture microdissection for the analysis of gastric cancer tissue. A 30-min nanoLC gradient facilitated the quantification of 2514 proteins from 0.2 mm^2^ regions representing cancer cells, cancer-associated fibroblasts (CAFs), and immune cells, as defined by multiplex immunohistochemistry (mIHC). This spatial proteomics approach revealed cellular heterogeneity within the tumor microenvironment of a clinical gastric cancer sample, and targeted validation identified the differential expression of 12 cancer-associated proteins comprising 103 peptides between cancer cell-enriched and CAF-enriched regions. This workflow offers a practical strategy for spatial proteomic analysis of clinical tissue samples.

## Highlights

• Evaluation of the Zeno TOF 7600 with nano-LC ensuring sensitivity and reproducibility.
• Image-based segmentation and navigation for mIHC FFPE tissue LCM.
• Cell type-resolved spatial proteomic profiling with improved efficiency.
• Targeted proteomics for spatially resolved protein analysis within the same workflow.

## Graphical Abstract

**Figure:**
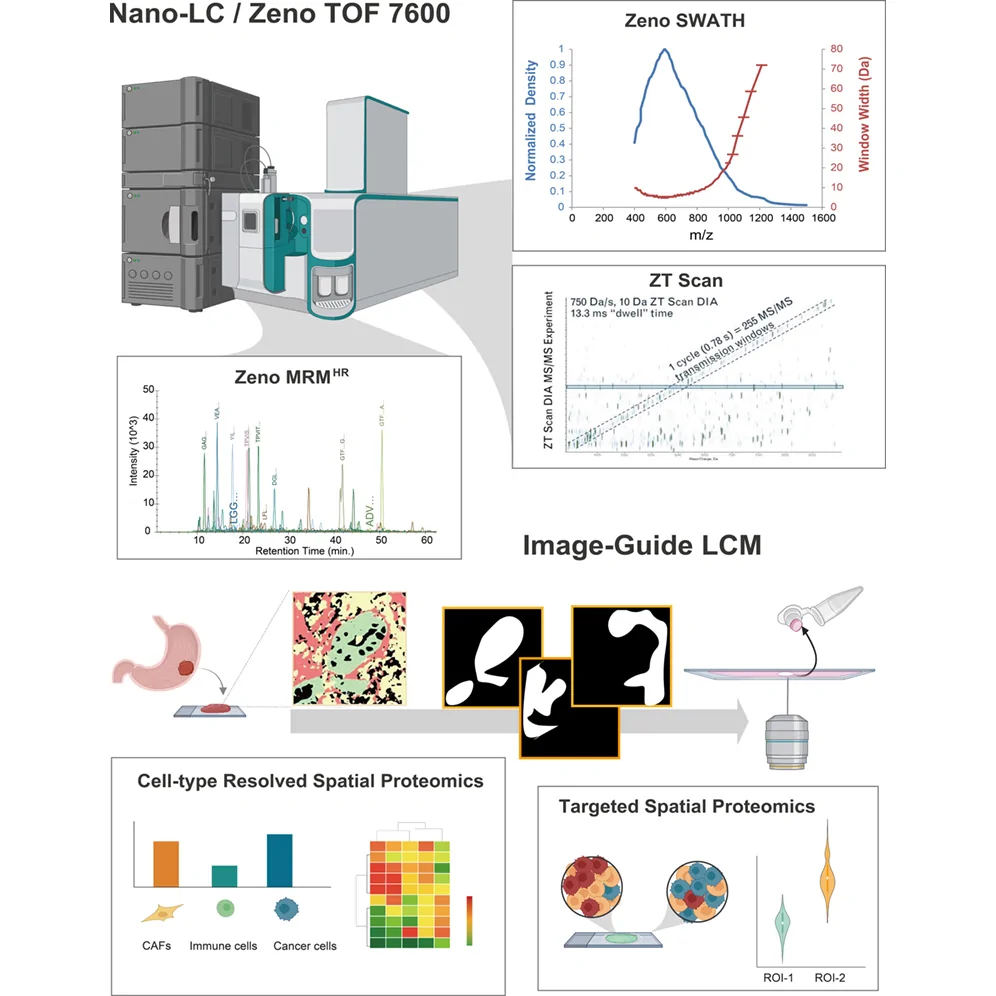

## In Brief

This study presents systematic evaluation for the performance of nanoLC coupled with Zeno TOF 7600 platform and applied to spatial proteomics. We establish an image-guided workflow that enables cell type–resolved protein profiling using data-independent acquisition (DIA) from laser micro-dissected clinical FFPE tissue samples. The performance and accuracy of the platform were further validated using a targeted mass spectrometry approach.

Spatial proteomics has received considerable attention in recent years 1, 2 due to its ability to reveal proteome heterogeneity in tissue samples (3), particularly in complex tumor microenvironments where cell type-resolved protein signatures are essential for understanding disease mechanisms (4). Additionally, liquid chromatography coupled with mass spectrometry (LC-MS/MS) has enabled several spatial cell type-resolved analyses, such as Deep Visual Proteomics (5) and the Spatial and Cell-type Proteomics (SCPro) workflow (6) by integrating with multiplex immunohistochemistry (mIHC) and laser capture microdissection (LCM). These approaches predominantly rely on nano-flow liquid chromatography (nanoLC) with advanced instrumentation design to achieve sensitive proteomic detection from limited tissue samples.

The Zeno TOF 7600 mass spectrometer incorporates Zeno trap technology, which releases ions sequentially from high to low m/z before they enter the TOF accelerator, enabling more efficient ion utilization and improved sensitivity (7). Studies using SWATH data-independent acquisition (DIA) have demonstrated substantial gains in proteome coverage and sensitivity with this platform. For instance, analytical-flow (>200 μl/min) LC coupled with Zeno SWATH has enabled biomarker discovery and pathway analysis in human and cynomolgus monkey tissues (8). Similarly, micro-flow (∼5 μl/min) LC setups have achieved identification of over 5000 protein groups from as little as 62.5 ng of K562 digest (9). Gu *et al*. demonstrated that combining narrow isolation windows with Zeno SWATH acquisition on the Zeno TOF 7600 significantly improves proteomic identification depth and quantification precision from complex biological samples compared to conventional SWATH approaches (10). While these investigations have demonstrated the potential of the Zeno TOF 7600 platform using analytical- and micro-flow LC, they have not evaluated its performance coupled with nano-flow (∼300 nl/min) LC, which remains the predominant approach for high-sensitive proteomic analysis. Given that Zeno SWATH DIA has already been shown to provide deep proteome coverage, the combination of nano-flow LC with the Zeno TOF 7600 is expected to deliver optimal performance for spatial proteomic analyses with the possibility that has yet to be systematically explored. Furthermore, a new DIA acquisition method ZT scan, also known as scanning SWATH (11), is another addition to the Zeno TOF platform that remains to be tested. Unlike conventional SWATH, which acquires MS/MS spectra across a series of fixed precursor isolation windows, scanning SWATH continuously sweeps the quadrupole across the precursor m/z range (e.g., 400–900 m/z) with a defined isolation width (e.g., 3–20 m/z) during acquisition. This sliding-window design introduces an additional precursor dimension that enables precise precursor–fragment correlation and improved deconvolution, thereby enhancing identification confidence and quantitative accuracy.

For applications in tissue samples, DIA-MS on Zeno TOF 7600 system was previously applied to a mouse model of acute kidney injury (12). Burton *et al*. achieved identification of 3945 protein groups, by generating a comprehensive kidney sample-specific spectral library using 120-min data-dependent acquisition of eight samples each in three replicates (24 runs in total). More recently, Humphres *et al*. (13) suggested that the performance gap between nano-flow and micro-flow LC has become minimal for many proteomic applications. They optimized a workflow with the following parameters: 5-min sample loading time and 30-min LC gradient for separation, 50 variable SWATH windows, and a 10 ms MS2 accumulation time. With sample loads as low as 400 ng, this workflow outperformed conventional 90-min gradients on the TripleTOF 6600, and enabled identification of ∼2000 protein groups from eight rat organs. In their subsequent work (14), an analysis of matched formalin-fixed paraffin-embedded (FFPE) and fresh-frozen (FF) rat tissues from four organs demonstrated that, using the same workflow with 200 ng peptide input, as many as 2403 and 3214 protein groups could be identified in the FFPE and FF samples, respectively.

In this study, we aimed to systematically evaluate the Zeno TOF 7600 coupled with a nanoLC platform for (i) untargeted profiling with DIA mode in Zeno SWATH and the new data acquisition mode ZT scan, and (ii) targeted proteomics with MRM^HR^ acquisition mode. By further integrating with our previously reported SCPro workflow (6) for spatial proteomic analysis, we established a cell type-resolved spatial proteomics approach optimized for the Zeno TOF 7600 platform and applied to clinical gastric cancer tissue samples. Through these studies, we aim to demonstrate the platform's broader applicability in spatial proteomics and facilitate its adoption across diverse biological and clinical contexts.

## Experimental Procedures

### Experimental Design and Statistical Rationale

This study focused on evaluating the performance of the nano-flow LC-coupled Zeno TOF 7600 system, in particular its profiling and targeted capabilities using various acquisition methods. In addition, the effectiveness of this platform for spatial proteomics applications was also tested.

For global untargeted profiling, we employed a label-free quantitative approach using Zeno SWATH and ZT Scan modes. K562 digest standards of 200 ng, 100 ng, 50 ng, and 20 ng were used as the initial stage to evaluate typical identification performance. Lower concentrations, ranging from 50 ng down to 1 ng were analyzed to assess sensitivity with LC optimization. To evaluate quantification accuracy, two sets of experiments were designed. First, a HeLa digest background was spiked with 1× and 2× amounts of yeast digest, representing low-abundance components (10% and 20% of the total sample), to assess whether the measured ratios accurately reflected the expected 1:2 ground truth in a complex matrix. Second, following a recent benchmark study (15), six sample sets of three comparison groups with proportions for Human, Yeast, and *Escherichia coli* (HYE) digests were set ratios at human 1:1, Yeast and *E. coli* at 3:2, 4:1, and 45:5, respectively. All assessments were performed with three technical replicates.

For targeted proteomics, we first assessed quantification accuracy using a simple peptide mixture standard containing 31 peptides. This standard comprised 20 peptides from the PepCalMix kit and 11 iRT peptides, spiked into 100 ng HEK 293T digest. We performed an initial 1:1 dilution, followed by two subsequent 1:1 dilutions of the resulting solutions, thereby establishing four concentration levels for each set: 4, 2, 1, and 0.5 fmol/μl for the PepCalMix peptides (reported in fmol/μl), and 0.4×, 0.2×, 0.1×, and 0.05× of the original stock for the iRT peptides. Next, we evaluate the multiplex capability using a mixture of 184 synthetic peptides with a dynamic range from 0.01 ng to 10 ng, spiked into a matrix of 100 ng yeast digest at three concentrations (5-fold, 10-fold and 20-fold dilution). All analyses were performed with three technical replicates. According to the MCP Targeted MS Guidelines, the targeted proteomics analyses performed in this study are classified as Tier 3.

For spatial proteomics applications, we analyzed region-specific and cell type–resolved samples using the standardized method described above. Each sample type was processed with three biological replicates (n = 3).

### Standard Samples and Tissue Samples

For calibration and quality control purposes, we utilized standard peptide mixtures including MS Synthetic Peptide Calibration Kit PepCalMix (SCIEX, P/N 5045759) and iRT (Biognosys, Ki-3002-1). Custom synthetic peptides were acquired from GeneScript for targeted analyses. Method evaluation was conducted using the SWATH Acquisition Performance Kit containing K562 cell digest (SCIEX, P/N 5045757). Additionally, laboratory-cultured samples including HeLa cells, *Saccharomyces cerevisiae* (yeast), and *E. coli* (E.coli) were prepared. HeLa cells were maintained in Dulbecco's Modified Eagle Medium (DMEM) supplemented with 10% fetal bovine serum (FBS) and 1% penicillin-streptomycin at 37 °C in a humidified 5% CO_2_ atmosphere. Cells were harvested at approximately 80 to 90% confluence by washing with ice-cold PBS, adherent HeLa cells were directly lysed by adding 1 ml lysis buffer (8 M Urea, 50 mM NH_4_HCO_3_, 1× cocktail). Yeast and E.coli were cultured in YPD and LB liquid medium respectively and were grown with shaking at their optimal temperatures at 30 °C and 37 °C respectively. When their OD600 are around 0.6 to 0.8, they were harvested by centrifugation (4000 g, 10 min, 4 °C) and washed with ice-cold PBS. Same lysis buffer was used with HeLa cells. Protein extraction and enzymatic digestion of these cultured samples were performed following the Filter-Aided Sample Preparation (FASP) protocol (16). All processed samples were subsequently subjected to LC-MS/MS analysis to comprehensively evaluate the performance characteristics of our experimental workflows.

### Hematoxylin and Eosin (H&E) and Multiplex Immunohistochemistry (mIHC) Tissue Slide Staining

Formalin-fixed, paraffin-embedded (FFPE) gastric cancer tissue sections with a thickness of 4 μm were used in this study. The sections were mounted on membrane slides (BayOmics#BO-018-01). Sections were floated in distilled water at 37 °C to unfold, mounted on slides, and dried at 42 °C for 45 min to overnight. Tissue slides were baked at 65 °C for 45 min before stanning. For H&E staining, deparaffinized in xylene for 5 min, and rehydrated through a graded ethanol series (100%, 100%, 95%, 90%, 80%, 70%, 5 min each). After rinsing in distilled water for 5 min, sections were stained with hematoxylin for 3 min, rinsed and differentiated in 0.5% HCl-75% alcohol for 2 s, blued in 0.2% ammonia for 10 s, and counterstained with eosin for 30 s. Following a final water rinse, sections were dehydrated through a graded ethanol series (70%, 80%, 90%, 100%, 60 s each, cleared in xylene for 60 s, and mounted with a synthetic resin. For mIHC, 4-color staining strategy using tyramide signal amplification (TSA) technology. Staining was performed using an automated staining machine (Dartmon, AS330-PLUS), which completed four rounds of staining to label primary antibody PanCK (CST#4545, 1:1000), αSMA (Proteintech#14395-1-AP, 1:2000), and CD45 (CST#70257, 1:500). Antigen retrieval was conducted using heat-induced epitope retrieval (HIER) in a pH 6.0 buffer at 100 °C for 20 min in each round. Primary antibodies were incubated for 60 min, followed by secondary antibody incubation for 30 to 45 min, and fluorescent dye staining was performed for 10 min in specific channels (520 nm for PanCK, 570 nm for αSMA, and 650 nm for CD45). Finally, DAPI staining was applied to all sections for nuclear visualization by incubating the slides in the dark at room temperature for 10 min, followed by mounting. The automated staining process ensured consistent and reproducible labeling across all samples.

### Imaging and Analysis

Tissue imaging was performed using the TissueFAXS imaging platform equipped with a 40× objective, achieving a resolution of 0.16 μm/pixel. Image analysis was conducted using TissueGnostics StrataQuest v7.1 software. A random forest algorithm was used to train the classifier for accurate identification of target features. Cancer cells, CAFs, immune cells were analyzed by applying specific gating thresholds to exclude false-positive cells and excessively small cells. To preserve intact cell morphology for subsequent laser capture microdissection (LCM), the mask was expanded by 15 pixels (0.16 μm/pixel) during the analysis process. VistaNavi software (BayOmics, version 1.0.2) was used to process scanned images, mask images, and XML files containing positional information (typically represented as rectangular frame paths from the LCM system). The software generated executable cutting-path XML files with adjusted scale and orientation. LCM was performed on a CellCut system (Molecular Machines & Industries, MMI) with a 20× objective. The laser parameters were set as follows: velocity: 40 μm/s, focus: 164 μm, power: 30%. Each dissection was repeated once.

### Sample Preparation

The collected LCM samples from gastric tissue slices were sonicated in 30 μl lysis buffer in the SISPROT PAC Kit (BayOmics, Catalog No. J18-01-000041) using non-contact sonication (Scientz08-3C) for 20 min (15 s on, 30 s off, 540 W) at 4 °C. Lysate was transfer to PAC tip for proteomics sample preparation following the manufacturer’s instructions, which is a commercial products originating from iPAC (6). Briefly, prior to sample loading, the PAC Tips were activated and equilibrated, after which an equal volume of tissue lysates was mixed with a loading buffer and loaded onto the tips. Proteins were reduced by incubation with reducing reagent DL-dithiothreitol (DTT) for 30 min at 37 °C. Subsequently, 6 μl digestion solution containing sequencing-grade trypsin was added to the tips and incubated for 3 h at 37 °C for digestion in the dark. The digested peptides were desalted and eluted into a glass injection insert and then lyophilized and reconstituted in 0.1% formic acid water prior to LC-MS/MS analysis. The samples comprised three biological replicates.

### LC-MS/MS

Nano-flow liquid chromatography (LC) analyses were performed using a M-Class II UPLC system (Waters) operated in the direct-inject mode. The mobile phases consisted of (A) 0.1% (v/v) formic acid in 98:2 (v/v) water/acetonitrile and (B) 0.1% (v/v) formic acid in 98:2 (v/v) acetonitrile/water. Chromatographic separations were conducted on a home-made column (75 μm I.D. × 15 cm, 1.9 μm C18) at a flow rate of 300 nl/min. Gradient elution protocols were applied with a mobile phase B range of 3 to 35%. For K562 dilutions, samples at concentrations of 200 ng/μl and lower were analyzed using gradient durations of 10, 20, 30, 45, or 60 min, with a 1 μl injection volume. ZT18 mixtures were analyzed under identical chromatographic conditions, employing a fixed 30-min gradient (3–35% B) and a 1 μl injection volume. Gastric cancer tissue samples were processed similarly but with a 3 μl injection volum. These conditions ensured consistent chromatographic resolution across all sample types. Data-independent acquisition (DIA) experiments were conducted using a Zeno TOF 7600+ hybrid quadrupole time-of-flight mass spectrometer (SCIEX) equipped with a horizontal nanoflow electrospray ionization (NSI) source Turbo V and OptiFlow interface. Standardized ion source parameters were applied across all experiments, including an ion spray voltage of 3800 V, curtain gas at 35 psi, nebulizing gas (Gas 1) at 18 psi, and an interface heater temperature of 175 °C. Three Zeno SWATH DIA acquisition methods were employed, each utilizing a Q1 mass range of 400 to 900 Da with different number of variable-width windows and MS/MS accumulation times 38 windows with 32 ms accumulation time, 65 windows with 20 ms accumulation time, and 85 windows with 18 ms accumulation time. All methods incorporated a TOF-MS precursor survey scan (400–1500 Da) with 200 ms accumulation time, collision-induced dissociation (CID) with dynamic collision energy ramping, and Zeno trap pulsing. The MS/MS mass range spanned 140 to 1750 Da. Triplicate acquisitions were performed for each experimental condition to ensure reproducibility. Additionally, ZT Scan DIA experiments were optimized for 30-min chromatographic gradients, using a peak width at half-height (PWHH) setting of >4.5 s. Data was acquired in triplicate for all samples under these conditions. The targeted mode was configured in MRM^HR^, using a 250 ms full-scan duration with the same scan range and source parameters as those set in the SWATH mode. Each MS2 scan was executed with an acquisition time of 15 ms, employing an optimized collision energy (CE) setting for enhanced fragmentation efficiency.

### Data Processing

For method evaluation by standard K562 cell digest, MS data was processed using DIA-NN (17) (version 1.8.16, https://github.com/vdemichev/DiaNN).The data was searched against a pre-constructed spectral library comprising K562 and HeLa cell line proteomes (SCIEX) with default settings. Briefly, the digestion enzyme was set to Trypsin/P. Up to one missed cleavage was allowed. Carbamidomethylation (C, +57.021 Da) was set as a fixed modification, and oxidation (M, +15.995 Da) and N-terminal acetylation (+42.011 Da) were set as variable modifications. The FDR threshold was 1% (q ≤ 0.01) at both precursor and protein levels. The precursor m/z range was set to 400 to 900, and the fragment m/z range to 140 to 1750. Mass accuracy tolerances were set to 20 ppm for MS2 and 12 ppm for MS1. For ZT Scan DIA analyses, data processing was optimized using the --scanning-swath parameter. Triplicate runs under identical conditions were processed independently.

For cell type–resolved spatial proteomic data, Spectronaut (v18.2.2, Biognosys) was used in hybrid DIA mode. Three cell-type samples with three biological replicates each were analyzed. Hybrid searches were performed using a spectral library generated from six fractions prepared from the same tissue type as the cell-type samples (adjacent tissue sections), and UniProt *Homo sapiens* database (UP000005640_9606_HUMAN, 20,406 entries). The digestion enzyme was set to Trypsin/P. Up to two missed cleavages were allowed. The mass tolerance was dynamically calibrated at ± 10 ppm for precursors and ± 20 ppm for fragments. Carbaminomethylation of cysteine was included as a fixed modification. Methionine oxidation was included as a variable modification. The FDR threshold was 1% (q ≤ 0.01) at both precursor and protein levels.

For targeted proteomic data, processing was performed using Skyline 23.1.0 with default peptide settings. The ion match tolerance was set to 0.2 m/z, allowing only precursor ions with charge states of +2 and +3, and fragment ions (b and y types) with a charge state of +1. Monoisotopic precursor and product ion masses were utilized, with collision energy parameters optimized for SCIEX instrumentation. MS1 filtering was set to "None" and the MS/MS acquisition method was configured to PRM, employing a resolving power of 30,000 through the TOF analyzer. All matching scans were included.

### Statistics Analysis

All statistical analyses were conducted using R software (R version 4.4.2) with packages including tidyverse, ggplot2, ggpubr, gridExtra, limma, dplyr, and pheatmap. Proteins were filtered to retain only those with at least three valid values in at least one experimental group. The raw intensity values were log2-transformed, and any missing values were replaced with zeros. Differentially expressed proteins between two groups were presented by scatter plot analysis, in which proteins are highlighted if their fold-changes are at least of four (in either direction), and the adjusted *p*-values were no more than 0.05. Differential expression analysis among three groups was performed using One-way ANOVA with Benjamini-Hochberg correction for multiple testing was performed on the filtered dataset. Proteins with adjusted *p*-values ≤0.01 were subjected to hierarchical clustering, using Pearson correlation as the distance metric and average linkage, resulting in a heatmap. Principal component analysis (PCA) was applied to assess variance and clustering among the studied groups. Gene Ontology (GO) enrichment analysis was performed using Metascape (18).

### Ethics Approval and Patient Consent Statement

Gastric cancer tissue sections were collected following the ethical approval (SYL-202124-02). All experiments were performed in accordance with the Declaration of Helsinki.

## Result

### Performance of Data-Independent Acquisition with Zeno SWATH and ZT Scan

Prior studies have shown that the implementation of SWATH DIA with the Zeno On mode demonstrated significant improvements in data acquisition performance compared to Zeno Off 8, 9. We first compared the number of identified precursors and matched b/y fragment ions between the two modes. SWATH analysis of a 200 ng standard K562 digest sample showed that the Zeno On mode attained greater number and intensity of precursor identifications compared with the Zeno Off mode (Fig. 1, *A* and *B*). Furthermore, although Zeno On SWATH acquired a slightly lower number of MS2 scans (242,250 for Zeno On versus 268,175 for Zeno Off) due to 2 ms Zeno Trap delay time for each cycle, it yielded a much higher number (about 8.8 times) of matched b/y fragment ions (Fig. 1*C*). This improvement resulted in a markedly higher fragment ion matching rate, defined as matched b/y ions divided by the number of acquired MS2 scans (1.748 for Zeno On compared with 0.180 for Zeno Off). Consequently, Zeno On SWATH identified 36.9% more protein groups and 64.4% more peptides than Zeno Off SWATH (Fig. 1*D*). In terms of the number of fragments per precursor, the Zeno On mode produced a greater number of matched b/y fragment ions per precursor (9.5) compared to Zeno Off (1.77). This implied that Zeno On was able to generate higher-quality MS/MS spectra, thereby enhancing the overall efficiency of peptide and protein identification. The same trend was observed for injection amounts spanning 20 to 200 ng of K562 digest samples (Fig. 1*D*).

**Fig.1:**
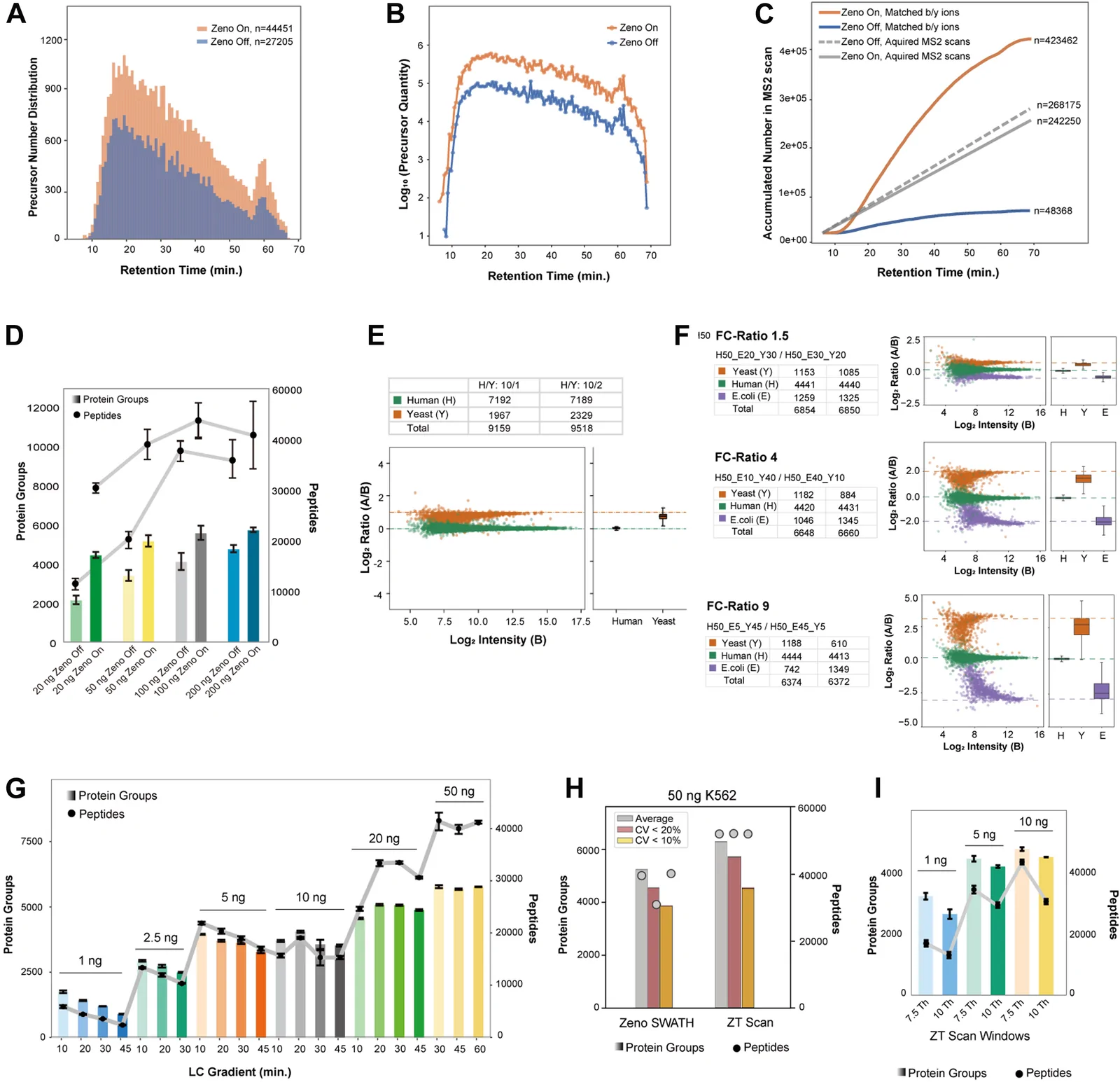
**The Performance of SWATH with Zeno Off and Zeno On Mode.***A*, identified precursor distribution across LC gradient in SWATH with Zeno On (*Orange*) and Zeno Off (*Blue*). *B*, quantified precursor intensities across LC gradient in SWATH with Zeno On (*Orange*) and Zeno Off (*Blue*). *C*, accumulation number in MS2 Scan. Acquired MS2 scan with Zeno Off (*Gray dot line*) and Zeno On (*Gray line*) and Numbers of matched b/y ions with Zeno Off (*Orange*) and Zeno On (*Blue*). *D*, number of identifications of 20 ng, 50 ng, 100 ng and 200 ng K562 sample injections with 3 species using SWATH with Zeno Off and Zeno On mode. *E*, protein identification and relative quantification ratio in a human-yeast mixture at 1/10 and 2/10 ratios, with a total of 200 ng per sample and three replicates. Each point represents an individual protein, and the *boxplot* shows the distribution of log_2_ ratio values for human (*green*) and yeast (*orange*). *F*, protein identification and quantification in a three-species mixture (human, yeast, and E. coli) at six different ratios, with a total of 100 ng per sample and three replicates. Ratios of 1.5, 4, and 9 are shown for each species. Each point represents an individual protein, and the *boxplot* shows the distribution of log_2_ ratio values for human (*green*), yeast (*orange*), and *E. coli* (*purple*). *G*, number of identifications from 1 ng to 50 ng K562 samples with three replicates, using Zeno SWATH under different LC gradients (mobile phase B range of 3-35%). *H*, number of identifications for 50 ng K562 samples using Zeno SWATH and ZT Scan. *Bar plot* represents protein group numbers with all (*grey*), CV < 20% (*Red*), and CV < 10% (*Yellow*). Dot plot represents peptide numbers with three replicates. *I*, number of identifications from 1 ng to 10 ng K562 samples using ZT Scan with 7.5 Th and 10 Th scanning modes.

To assess Zeno SWATH quantification accuracy, we analyzed 200 ng samples of HeLa and yeast protein digests mixed at 1:10 and 1:5 ratios. Zeno SWATH accurately quantified 1967 and 2329 yeast protein groups respectively in these mixtures where yeast proteins represented only a minor fraction of total proteins (Fig. 1*E*). This precision was achieved while maintaining extensive proteome coverage, with 9518 total protein groups identified. To validate quantitative performance with more complex samples, we analyzed six multi-species samples containing defined proportions of digested HeLa, yeast, and *E. coli* (15). Each sample contained a total protein amount of 100 ng and was acquired using Zeno SWATH for relative quantification across species. The distribution of protein quantification ratios (Fig. 1*F*) closely mirrored with the expected mixing proportions across all samples. To assess Zeno SWATH performance with limited sample input , we evaluated the effect of shortened chromatographic gradients on protein and peptide identifications (Fig. 1*G*). For samples below 5 ng, shortened gradients significantly improved protein and peptide identifications. This improvement might be attributed to the generation of sharper elution peaks, which enhanced detectability through increased signal intensity. This finding is in line with previous low-input studies, which have also reported that shorter gradients can enhance identification rates compared to longer ones 19, 20, 21. Using a 10-min LC gradient (mobile phase B range of 3–35%), we identified 1820 protein groups and 6146 peptides from 1 ng K562 digest and 3989 protein groups and 22,342 peptides from 5 ng input. Notably, our 2.5 ng results yielding 2999 protein groups and 13,596 peptides, surpassing the best microflow-LC performance in the literature (9), likely due to the sensitivity of nano-LC at low sample inputs.

Additionally, we compared ZT Scan, also known as scanning SWATH (11), with Zeno On SWATH in replicate analyses of 50 ng K562 digest (Fig. 1*H*). ZT Scan showed improved reproducibility, with more proteins exhibiting low variability across replicates. Identified protein groups increased from 6491 to 7141 and peptide identifications from 44,290 to 61,026. The percentage of proteins with CV values below 20% and 10% improved by 24.2% and 45.7% respectively, while the improvements were 55.8% and 86.3% at the peptide level (Supplemental Fig. S1). When comparing 7.5 Th and 10 Th scanning windows under a 30-min LC gradient (Fig. 1*I*), the 7.5 Th window consistently yielded more identifications across all sample inputs. With 1 ng of K562 digest, the 7.5 Th window identified 3142 protein groups and 17,367 peptides, comparable to Zeno SWATH results with a larger input (5 ng) under identical conditions. The improvement became less pronounced at higher sample inputs (5–50 ng), suggesting that the ZT Scan mode was especially effective under low-input conditions.

### Targeted Proteomics Evaluation Using Zeno MRM^HR^

We evaluated the performance for targeted proteomics using 11 iRT peptide using MRM^HR^ method. We allocated 250 ms for MS1 full scan and 10 ms per peptide for MS2 scan. This 10 ms accumulation time produced well-defined peaks, with minimal signal improvement when extended to 100 ms (Supplemental Fig. S2*A*). The Zeno On mode significantly enhanced peptide signal intensity. All 11 iRT peptides across various retention times and concentration ranges showed >5-fold signal enhancement under Zeno On compared to Zeno Off conditions (Fig. 2, *A* and *B*). Individual fragment ions in Zeno Off mode displayed signal intensities below 1000, potentially compromising detection in complex samples due to background interference. Conversely, Zeno On mode elevated these signals to over 7000, substantially improving fragment ion matching and enabling quantification of lower-abundance peptides (Fig. 2*C*).

**Fig.2:**
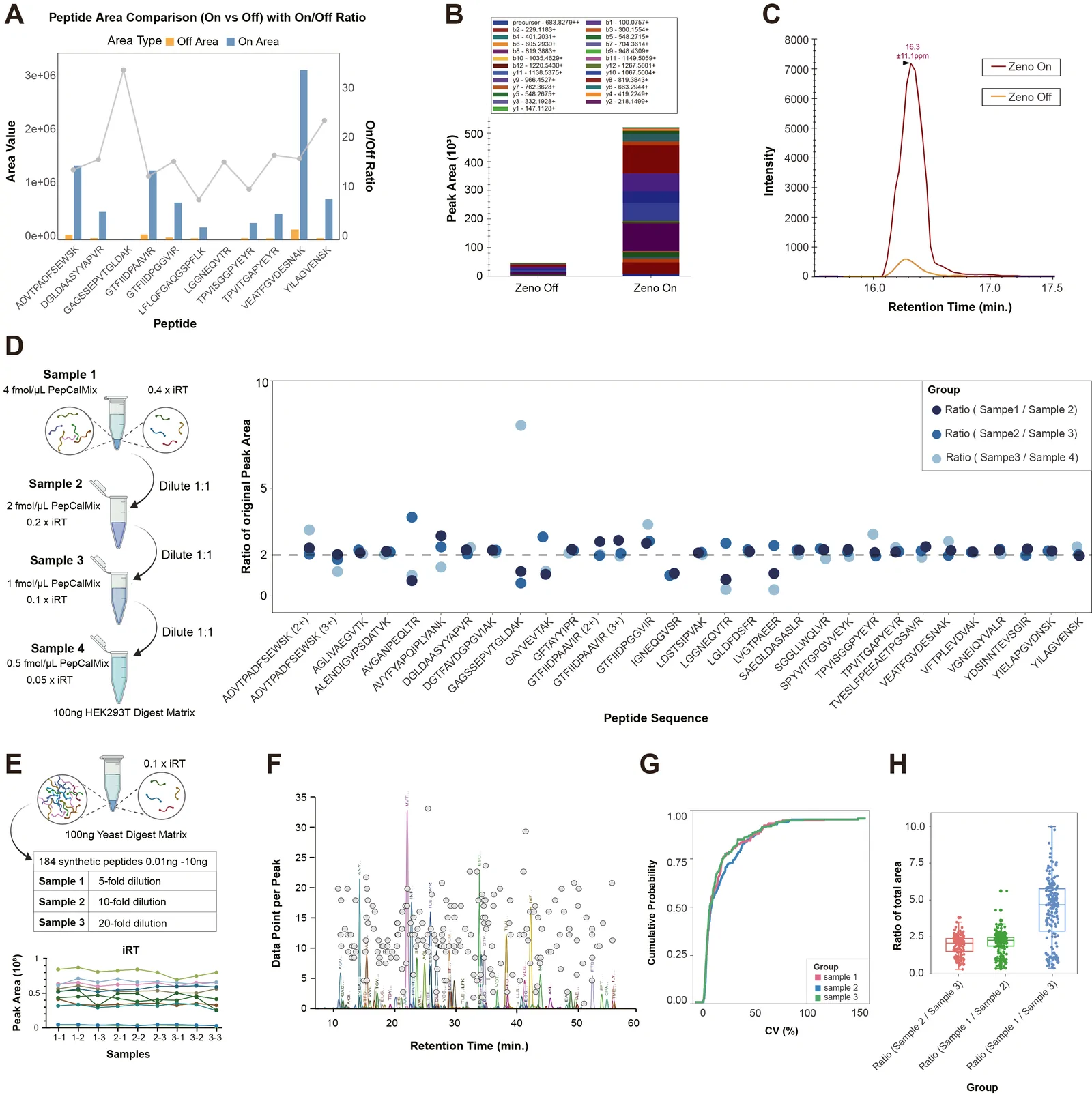
**Performance of MRM^HR^ with Zeno Off and Zeno On Mode.***A*, peak area and comparison ratio of 11 iRT peptides detected using MRM^HR^ with Zeno On and Off mode. Each bar presents peak area of each peptide and point line presents ratios by comparing Zeno Off with Zeno On. *B*, peak area of a peptide accumulated with all fragment ions detected using MRM^HR^ with Zeno On and Off mode. *C*, intensity comparison of a fragment ion detected using MRM^HR^ with Zeno On and Off mode. *D*, sample setting by serial dilution of iRT and PepCalMix standard peptide mixtures spiked in 100 ng HEK 293T digest; Quantitative ratios at three comparison groups representing concentration levels. *E*, sample setting of 184 synthetic peptides spiked in 100 ng yeast digest as matrix with equal amount of 0.1 × iRT for each diluted sample; iRT peptide peak areas across three groups with three replicates shown below. *F*, the number of data point per peak of each precursor over LC gradient. *G*, CV (%) of total detected peptides in three different concentration groups. *H*, the distribution of total area ratios among 3 comparison groups in different concentrations.

Next, to assess quantitative accuracy, a mixture of 31 peptides was prepared by combining 11 iRT peptides (at 0.4 × of original concentrations of the product) and 20 PepCalMix peptides (at 4 fmol/μl). This mixture was then spiked into a matrix consisting of 100 ng of HEK 293T lysate and subjected to 1:1 serial dilution, generating four concentration points (Fig. 2*D*). Quantitative analysis revealed that for most peptides, the measured ratios closely matched the expected 2-fold change between successive dilution points. For example, peptide ADVTPADFSEWSK deviated from the expected ratio in low-concentration samples, showing divergent quantification between its 2+ and 3+ charge states, whereas it maintained the expected ratios at medium to high concentrations. Peptide DGTFAVDGPGVIAK exhibited consistently accurate abundance ratios across all comparing groups, supported by high-quality peak extraction (Supplemental Fig. S3). Conversely, peptide GAGSSEPVTGLDAK displayed a significant outlier in the low-concentration group, likely due to sample matrix interference, and exhibited ratio compression at higher concentrations attributable to a missing peak in one replicate. Additionally, hydrophilic peptides eluting early in the gradient were more susceptible to signal loss, a phenomenon potentially exacerbated by adsorption effects amplified by the 3-day interval between replicate acquisitions.

One of the primary challenges in implementing routine targeted proteomics lies in the complexity of the sample matrix and limitations in dynamic range, which complicates the development of optimized methods for quantifying numerous peptides simultaneously. To assess the Zeno platform for targeted proteomics, we attempted to measure 184 synthetic peptides ranging from 0.01 ng to 10 ng prepared in three dilutions of 5-fold, 10-fold, and 20-fold spiked into 100 ng Yeast digest, with 0.1× iRT peptides as equal-amount quality control. Results showed consistent detection of iRT peptides across all samples and replicates as illustrated in Figure 2*E*. Using a 60-min nano LC gradient with unscheduled target MS, we detected 161 out of 184 peptides, achieving a 93% detection rate, with sufficient data points per peak (over 10 on average) for quantification (Fig. 2*F*). Over 70% of the detected 161 peptides in three replicates in each sample achieved quantification coefficient of variation (CV) below 20% (Fig. 2*G*). Relative quantification between sample groups revealed that median abundance ratios were consistent with the expected ratios (Fig. 2*H*), indicating accurate peptide quantification despite complex matrix background. These results validate our targeted proteomics workflow for reliable quantification of multiplex targets with wide dynamic range.

### Implementing Spatial Proteomics Using Zeno TOF 7600

Building on the demonstrated capabilities of the nano-LC coupled Zeno TOF 7600 system for both deep proteomic profiling and targeted multiplexed analysis, we extended its application to a spatial proteomics workflow using FFPE clinical gastric cancer samples. Histological and molecular characterization was performed using hematoxylin and eosin (H&E) staining and mIHC. Tissue sections were processed using LCM to isolate specific regions of interest, followed by SISPROT sample preparation (Fig. 3*A*).

**Fig.3:**
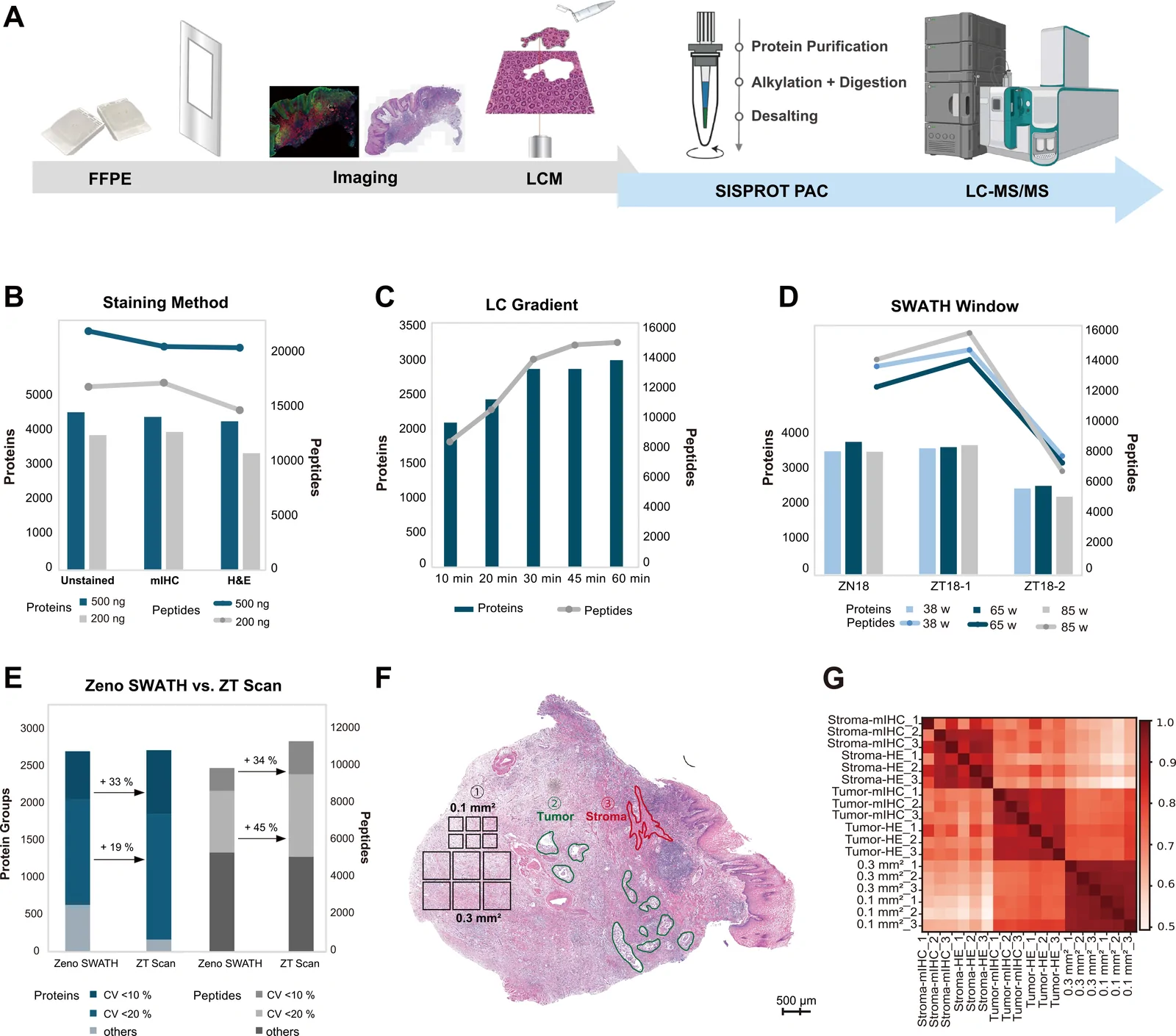
**Implementation of spatial proteomics workflow for FFPE tissue samples.***A*, Workflow of spatial proteomics analysis for FFPE tissue samples. Tissue sections were stained with H&E or mIHC, followed by laser capture microdissection (LCM) to collect regions of interest (ROI), which were then subjected to SISPROT sample preparation for LC-MS/MS. *B*, comparison of protein and peptide identification numbers at 200 ng and 500 ng from LCM tissue samples processed under three conditions: unstained, mIHC-stained, and H&E-stained. *C*, LC gradient optimization using 0.2 mm^2^ ZT18-2 LCM samples. Each injection corresponds to protein amount from 0.2 mm^2^ area, while each sample was derived from multiple 0.2 mm^2^ tissue. *D*, evaluation of protein and peptide identification numbers under different SWATH window settings (38, 65, and 85 windows) on 0.2 mm^2^ LCM samples with 3 sample types. *E*, comparison of peptide identification numbers of ZT18-2 acquired by Zeno SWATH and ZT Scan under CV < 20% (*Darker blue*) and CV < 10% (*Grey*). *F*, overview of H&E-stained ZT18-2 and Region of Interest for each cell type in black lines. ① square area: 0.1 mm^2^ and 0.3 mm^2^, ② Tumor-enriched region, ③ Stroma-enriched region, with collection area of 0.1 mm^2^ for each sample. *G*, correlations among 3 types of samples stained with H&E and mIHC, with 3 replicates of each stained sample. The color gradient (*light* to *dark red*) represents correlation coefficients from 0.5 to 1.0.

Next, we went on to investigate different parameters that affect the sensitivity of spatial proteomic analysis. Comparative analysis for staining methods revealed that both H&E and mIHC staining methods yielded comparable protein and peptide identification numbers to unstained sections, which identified 3705 and 4331 protein groups and 17,567 and 22,691 peptides at 200 ng and 500 ng injection levels, respectively (Fig. 3*B*). This demonstrates that staining does not significantly compromise protein identification. We then optimized the workflow using a tissue area of 0.2 mm^2^ to reflect realistic sample limitations in clinical settings. Our gradient optimization showed that a 30-min effective gradient produced 2859 protein groups and 13,688 peptides. This represented only a 10% reduction in peptide yield comparable to the 60-min gradient, while the throughput is doubled (Fig. 3*C*). Evaluation of SWATH acquisition window settings across three distinct tissue regions demonstrated minimal differences in protein identification numbers between 38, 65, and 85 variable windows (22) (Fig. 3*D*), indicating marginal effect of window configuration on identification performance. Furthermore, ZT Scan for a 0.2 mm^2^ sample resulted in 2722 identified protein groups, similar to Zeno SWATH (2707 identified protein groups). However, ZT Scan achieved more stable CVs, especially at the peptide level (Fig. 3*E*), making it the preferred mode for consistent and reliable spatial proteomics analysis.

Finally, to demonstrate the utility of spatial proteomics to gain additional biological insights, tumor-rich and stroma-rich regions were manually identified using H&E staining (Fig. 3*F*) and mIHC (Supplemental Fig. S3*B*). Two different sampling areas (0.1 mm^2^ and 0.3 mm^2^) were compared. Analysis revealed significant correlation differences between tumor-rich and stroma-rich regions, confirming their distinct proteomic profiles. Conversely, the 0.1 mm^2^ and 0.3 mm^2^ samples of the same region showed high correlation, reflecting the relative homogeneity within regions (Fig. 3*G*). Strong correlations were observed between mIHC fluorescence and H&E staining data from identical sample types (Supplemental Fig. S3*C*). The distinct proteomic signatures between tumor and stromal regions, coupled with the consistency within each cell type, underscore the importance of cell type-resolved analysis for accurate characterization of tumor microenvironments.

### Image-Guided Cell Type–Resolved Segmentation and Dissection

For cell type-resolved spatial proteomics sample collection, we performed four-color mIHC staining using DAPI, pan-CK, α-SMA and CD45 to label the cell nuclei, cancer cells, cancer-associated fibroblasts (CAFs) and immune cells, respectively. Panoramic screening of whole-slide images identified a tissue region of interest (ROI) of 8.43 mm^2^ containing all cancer cells (Fig. 4*A*). Using the Classifier module of the StrataQuest imaging analysis system to automatically identify positive shapes, we first labeled representative cancer cells as positive examples and other regions as negative examples to train a random forest model that generated cancer cell shape masks (Fig. 4*B*). Then, we refined the segmentation by excluding DAPI-negative cells, areas smaller than 1500 μm^2^, and cells with marker signal intensities below 100 arbitrary units. Histogram-based gating was performed to select tumor regions within specific size ranges, yielding 45 tumor-positive shapes (Fig. 4*C*). The finalized masks were processed with VistaNavi software to generate cutting-path XML files for the LCM system, with paths manually adjusted to ensure precise alignment with tissue morphology (Fig. 4*D*). Post-dissection fluorescence microscopy confirmed the integrity and specificity of collected samples on a sticky cap (Fig. 4*E*). In total, a tissue slide containing three distinct cell types with a collection area of 0.2 mm^2^ per type for three replicates, were successfully obtained for subsequent proteomic analysis.

**Fig.4:**
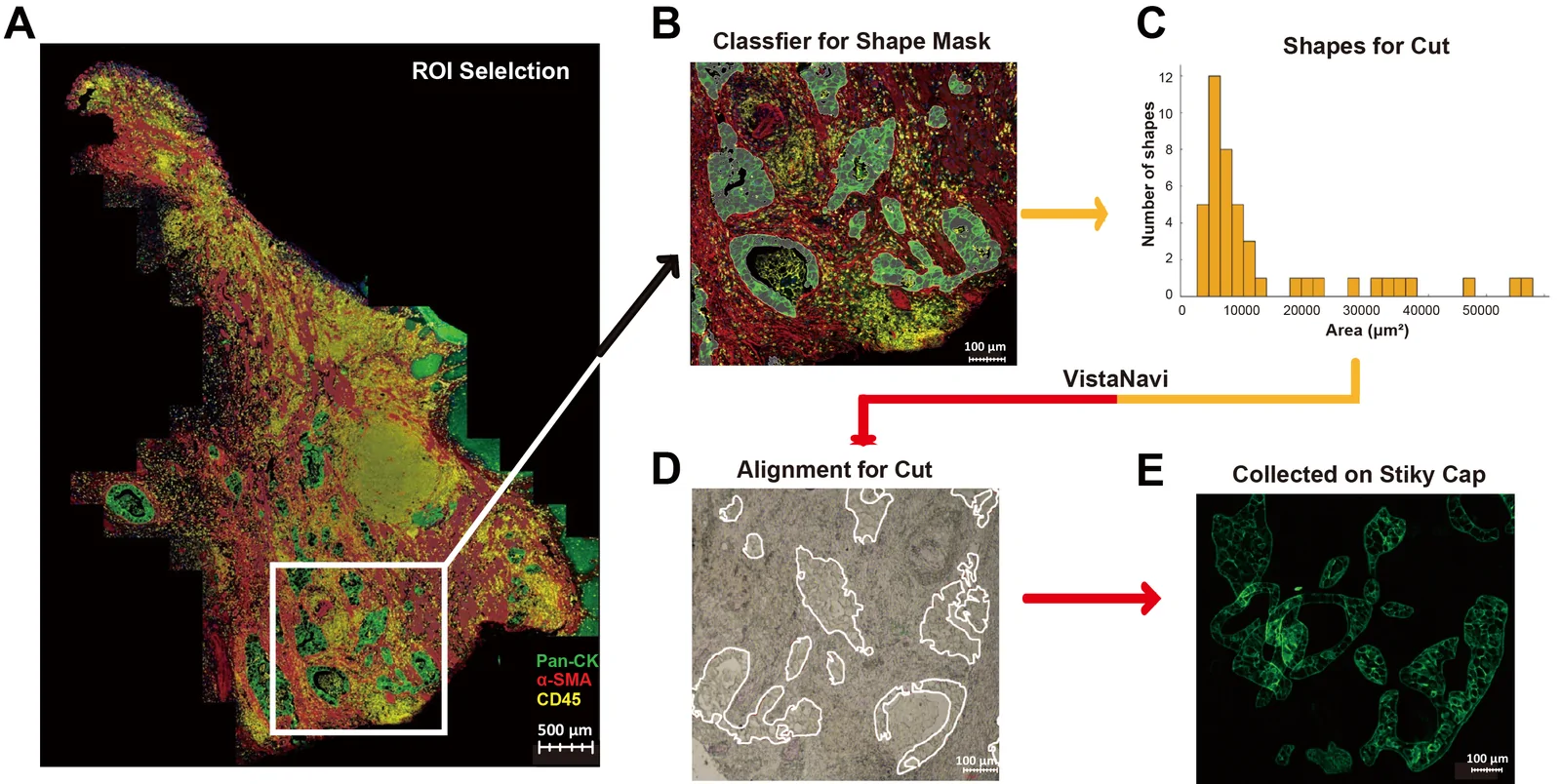
**Cell type-resolved spatial proteomics sample collection based on VistaNavi.***A*, ROI selection for the ZT18-2 sample, encompassing all tumor regions based on mIHC staining, cancer cell marker Pan-CK (*green*), α-SMA for CAFs (*red*), CD45 for immune cells (*yellow*) and DAPI (*blue*) for nucleus. *B*, cancer cell mask (*white outline*) generated using a trained random forest classifier and iteratively refined to remove misclassified regions. *C*, histogram-based gating to select cancer cells within biologically relevant size ranges. *D*, alignment of the XML cutting path (*white lines*) generated by VistaNavi software with real-time tissue morphology on the laser capture microdissection platform. *E*, fluorescence validation of the collected cancer cell shapes on a sticky cap.

### Cell Type-Resolved Spatial Proteomics for a Patient With Clinical Gastric Cancer

Following our spatial proteomics workflow, we collected 0.2 mm^2^ tissue samples from cancer cells, CAFs, and immune cells. Microdissected samples were prepared using SISPROT PAC, specifically iPAC (6), which effectively cleaned spatial membrane-based LCM tissue samples. We quantified 2514 protein groups across 3 cell types, each with three biological replicates (Fig. 5*A*). Proteomic analysis of cancer samples revealed a dynamic range over four orders of magnitude in protein abundance (Fig. 5*B*). In cancer cell samples, KRT8 (23), a recognized gastric cancer marker, displayed significantly a high expression level. VIM, known as a surface marker of circulating tumor cells from gastric cancer (24), was also readily detected. Notably, TGFB1, a protein associated with gastric cancer (25) and chemotherapy response (26) in spatial omics studies, was detected at relatively low abundance. And the highest TGFB1 expression among 3 cell types was in CAFs samples (Supplemental Fig. S5*A*). This is consistent with prior knowledge that TGFB1’s activity is concentrated in CAF-enriched stromal regions (27). The detection of TGFB1 demonstrates the sensitivity and precision of our technology in capturing heterogeneous expression patterns of key regulatory proteins. Principal component analysis (PCA) showed clear separation among cancer cells, CAFs, and immune cells (Fig. 5*C*), indicating distinct proteomic profiles for each cell type and confirming the reliability of our spatial segmentation and cell type-resolved sampling process. A comparative analysis of differentially expressed proteins (DEPs) between cancer cells and CAFs, as well as between cancer cells and immune cells, revealed distinct expression profiles highlighting cancer-specific protein alterations (Fig. 5*D*). Proteins significantly upregulated in cancer regions compared to both CAFs and immune cells (Both Up, marked in red) included established cancer markers such as KRT8, KRT18, KRT19, S100P, and ANXA4, as well as GOLM1, ACSL6, ASS1, and SLC25A1. Conversely, proteins downregulated in cancer regions (Both Down, shown in blue) included LRRC45, COL8A1, PLCB2, ELN, WNT2B, SRPX, HIST1H1C, and SNTB2. In other words, these proteins exhibit cancer-specific expression patterns that can potentially define cellular identities within the tumor microenvironment. Among them, the known gastric cancer marker KRT8 demonstrated consistently high expression in cancer samples using both MS1- and MS2-based quantification methods (Fig. 5*E*). Between immune cells and CAFs, 513 proteins were differentially expressed with 211 upregulated in immune cells (Supplemental Fig. S5*B*), including immune-specific proteins such as CD74, HLA-class I and II proteins, and T-complex protein-1. Histone proteins were also significantly upregulated, likely due to the larger nuclei and higher nuclear content of immune cells.

**Fig.5:**
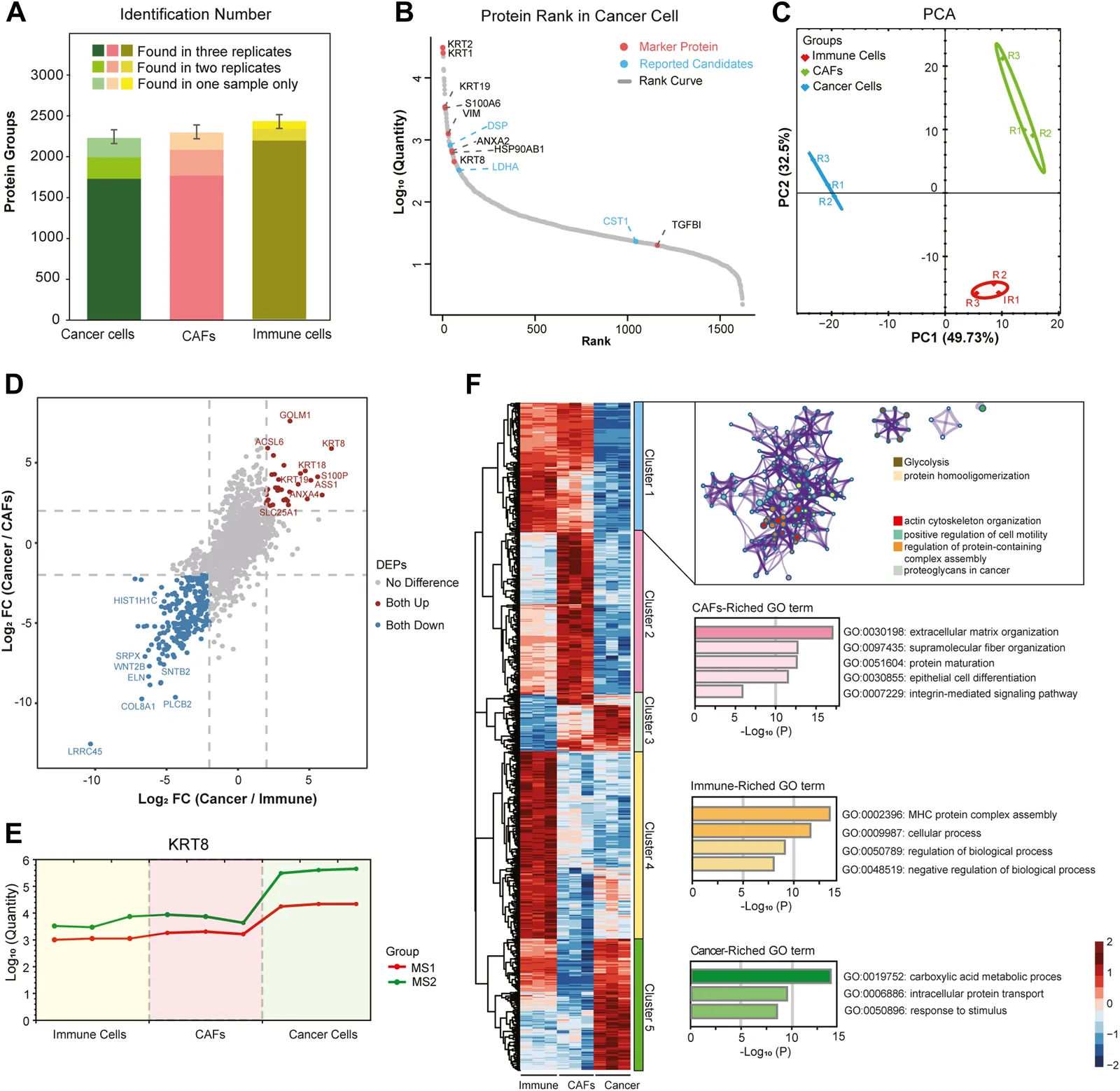
**Cell type-resolved spatial proteomics.***A*, protein group identification number in three cell type-resolved LCM samples. Data completeness presented with protein groups quantified within three to one replicate. *B*, cancer cell protein rank. Marker proteins (*red*) and proteins reported in the literature to be associated with gastric cancer (*blue*) are marked. *C*, PCA plot of three groups: Immune cells (*red*), CAFs (*green*), Cancer cells (in *blue*). PC1 (49.73%) and PC2, (32.5%) define the principal component space. *D*, scatter plot for differential protein expression between two comparisons, Cancer vs Immune cell type samples and Cancer vs CAFs with log_2_ (fold change). *E*, gastric cancer marker KRT8 Quantity in MS1 and MS2. *F*, Heatmap generated by one-way ANOVA followed by post-hoc tests. Each cluster was subjected to GO term enrichment analysis. Cluster 1 displays pathways and interaction networks. Clusters 2–5 show pathway enrichment and significance, except Cluster 3, which is not shown.

A heatmap of proteomic data showed clear clustering of the 3 cell types across five distinct clusters (Fig. 5*F*). Cluster one included proteins upregulated in both CAFs and immune cells but downregulated in cancer cells, with GO term enrichment in actin cytoskeleton organization, cell motility, and protein-complex assembly. Cluster two showed significant enrichment of upregulated proteins in CAFs, with functional annotations highlighting extracellular matrix (ECM) organization, supramolecular fiber organization, protein maturation, epithelial cell differentiation, and integrin-mediated signaling. Key ECM-related proteins in this cluster included COL4A, COL6A, COL1A families, ITGB1, FBLN3, and LAMA4, supporting the role of CAFs as stromal regulators. EFEMP1 in this cluster, quantified with a significant fold-change among three groups, has been implicated in gastric cancer progression (28). Cluster four contained proteins enriched in immune cells, including LMP2, a subunit of the immunoproteasome (29), with functions related to MHC protein complex assembly and various cellular regulatory processes. Cluster five comprised proteins predominantly expressed in cancer cells, involved in carboxylic acid metabolism, intracellular protein transport, and stimulus response. These findings demonstrate the functional heterogeneity within the gastric cancer microenvironment and highlight the distinct molecular signatures of cancer cells, CAFs, and immune cells. The consistent and biologically relevant protein expression patterns observed across cell types confirm the reliability of our workflow for cell type-resolved spatial proteomics.

### Region-Specific Targeted Proteomic Assessment

To evaluate the applicability of targeted proteomics for spatially resolved clinical samples, we selected a cluster of proteins highly correlated with gastric cancer marker KRT8 (Fig. 6*A*) as targets, resulting 117 peptides from twelve proteins were generated from the cell type-resolved proteome data without filtering. Two distinct ROIs were set: a cancer-enriched region (38.1% cancer cells), and another CAFs-enriched region with only 1.3% of cancer cells (Fig. 6*B*). The targeted approach successfully detected 88% of peptides (103/117). All 14 KRT8 peptides detected by ZT Scan untargeted profiling were likewise detected by MRM^HR^, with consistently higher expression in the cancer-enriched sample (Fig. 6*C*). While proteins like KRT18 were quantified using multiple peptides, others such as SLC25A6 relied on a single peptide quantification (DFLAGGIAAAISK). The targeted methodology optimized chromatographic peak quality and area, yielding reproducible signals across samples (Fig. 6, *D* and *E*), though SLC25A6 showed minimal expression differences between regions. Quantification of all 12 proteins revealed significant differential expression patterns (Fig. 6*F*). Besides KRT8, several proteins exhibited substantial enrichment in cancer regions compared to CAFs-enriched regions: ASS1 (4.3-fold), KRT18 (2.3-fold), TXRD1 (3.0-fold), ANXA4 (2.1-fold), and ALDH2 (2.2-fold). These results demonstrate the efficacy of targeted proteomic approaches for spatial samples and their potential utility for precise quantitative analysis in specialized clinical applications.

**Fig.6:**
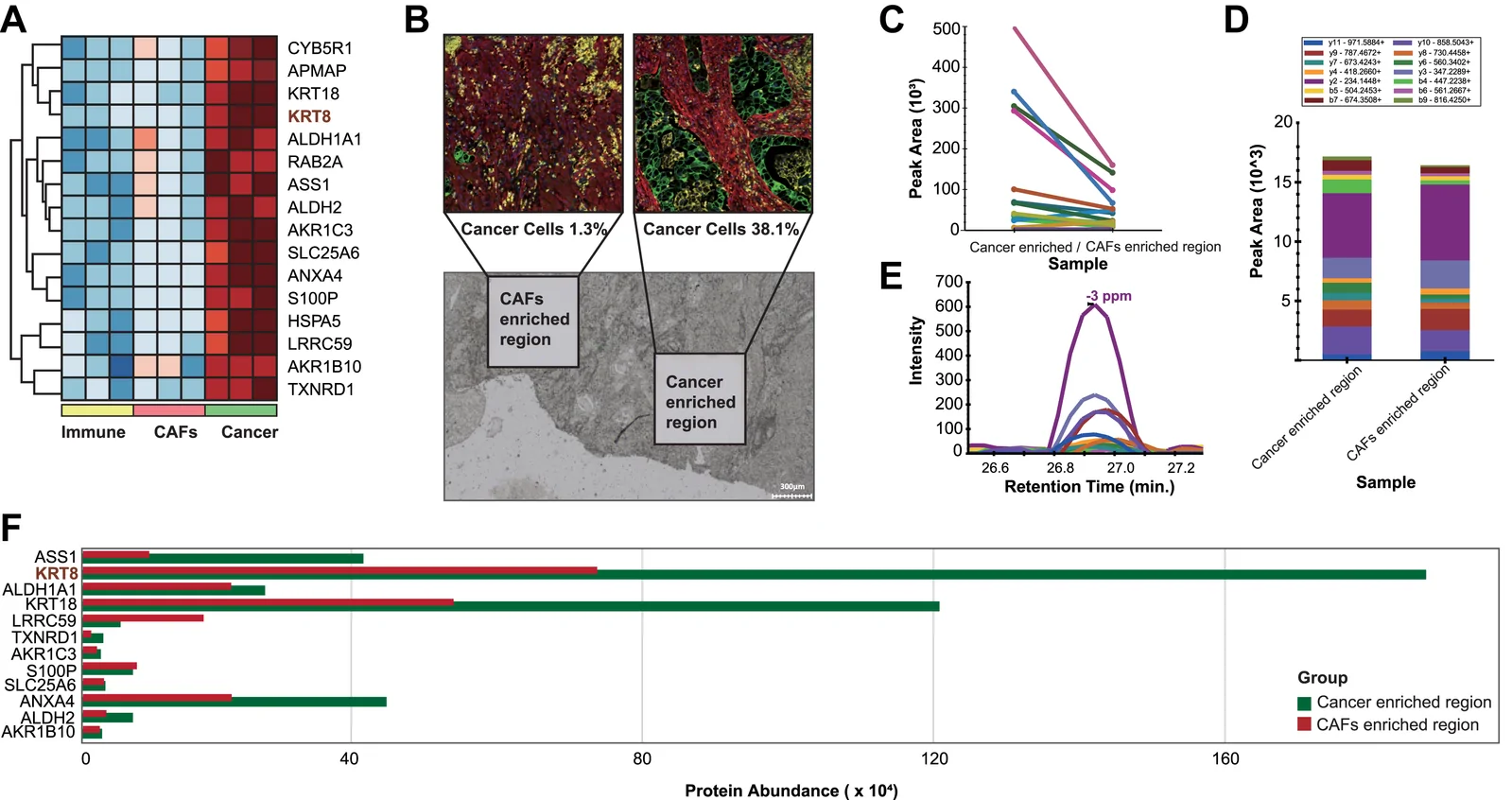
**Targeted spatial proteomics.***A*, selection of cluster proteins with gastric cancer marker KRT8. *B*, selected ROIs for targeted spatial proteomics: CAFs-enriched region containing minimal cancer cells content of only 1.3% and Cancer-enriched region with cancer cells content of 38.1%. *C*, quantification of 14 peptides of KRT8. *D*, quantification of single peptide DFLAGGIAAAISK for protein SLC25A6 and its expression levels in Cancer enriched region and CAFs enriched region. *E*, chromatographic transition peak quality of the of the only peptide DFLAGGIAAAISK for protein SLC25A6. *F*, protein abundance of 12 proteins in the Cancer-enriched region and CAFs-enriched region.

## Discussion

LC-MS/MS-based spatial proteomics, when combined with laser-capture microdissection, enables selective enrichment of specific tissue regions for proteomics analysis (30). The additional integration of mIHC imaging at cell-type resolution further allows precise isolation of marker-positive areas (6). However, existing studies (9) face challenges related to limited accessibility, specialized equipment and labor-intensive manual procedures that demand high precision. Several emerging technologies, such as novel MS acquisition modes and software for alignment image analysis with LCM, have yet to be systematically evaluated. In this study, we evaluated a Zeno TOF 7600 mass spectrometer coupled with nano-flow LC as a robust and integrated platform, enabling cell type-resolved proteomics profiling and validation in clinical FFPE tissues. By implementing image-guided laser microdissection sampling, combined with optimized data-independent acquisition (DIA) workflows, we successfully resolved proteomic heterogeneity within gastric cancer microenvironments. The optimized DIA workflow using a K562 standard digest as a test sample enabled the identification of over 7000 protein groups from nanogram-scale inputs. Although the number of identifications is comparable to that achieved with micro-flow LC at an input of approximately 50 ng, indicating no significant sensitivity advantage for nano-LC at this loading amount, nano-LC demonstrates a marked advantage at input amounts below 10 ng. This is particularly the case with the ZT Scan mode, which enabled the detection of 4 to 5 times more precursors compared to the leading report (9). Additionally, ZT Scan mode further improved reproducibility, with 45.7% of proteins with CV <10%, thereby addressing the variability issues inherent to spatially restricted samples. Targeted analyses validated the capability of the Zeno TOF 7600 to reliably quantify 184 peptides with an average of over 10 data points per peak, thus demonstrating considerable throughput alongside high precision.

In addition to its quantitative improvements, the use of a 75 μm i.d. column operated at 300 nl/min effectively balances sensitivity with robustness, advantages overcoming the low sensitivity issue operated at analytical-flow and micro-flow. The improved stability of this configuration facilitates prolonged operation and consistent data quality. Moreover, the system integrates high-resolution global proteome profiling via DIA with targeted analyses such as MRM^HR^, enabling a unified discovery-to-validation workflow within the same instrument. Importantly, the nanoflow-ZenoTOF 7600 is optimized for cost-effective, accessible spatial proteomics and can be implemented on widely available LC–MS configurations. Collectively, these features define a workflow that balances analytical depth, throughput, and practical feasibility, thereby expanding the accessibility of spatial proteomics research.

Using this platform, image-guided LCM sampling of gastric cancer tissues enabled quantitative analysis of cancer cells, CAF and immune cell-enriched regions, revealing distinct proteomic profiles that underscore the molecular heterogeneity of the tumor microenvironment. Furthermore, targeted analysis showed that the gastric cancer marker KRT8 was enriched in tumor regions, while stromal compartments (comprises CAF and immune cells) exhibited significant enrichment of extracellular matrix. These findings demonstrated the capability of this platform to perform cell type-resolved proteomic features, providing critical insights into tissue-specific protein expression and cellular heterogeneity. Furthermore, the workflow is compatible with FFPE samples and expands its applicability to retrospective clinical studies, offering a valuable tool for translational research.

Despite these strengths, challenges remain that warrant further optimization. Matrix effects observed in low-concentration targeted analyses highlight limitations in dynamic range, which may affect the detection of low-abundance proteins in complex samples. For spatial proteomics, smaller sampling areas may better preserve spatial information, such as the association between immune cells at varying distances from the tumor and therapeutic outcomes in gastric cancer (31). To probe spatial heterogeneity at the cellular level would require targeting specific cell types within a defined proximity, leading to inevitably smaller sample area and lower input for proteomics analysis. In this study, the compressed gradients did not achieve the optimal levels typically required for high-throughput or high-sensitivity workflows. Shortened chromatographic gradients improve throughput but result in reduced peptide yield compared to longer gradients, reflecting trade-offs in experimental design. Therefore, employing sample preparation methods that minimize sample loss, along with low internal diameter (I.D.) LC columns, is advantageous for maintaining proteomic depth in these small-scale samples (32).

In summary, the Zeno TOF 7600 coupled with nano-LC workflows offers a transformative tool for spatial proteomics, enabling high-resolution analysis of limited clinical samples and providing critical insights into cellular heterogeneity and tumor microenvironments. Looking forward, the recently introduced Zeno TOF 8600 system promises significant further advancements up to 10-fold improvement in MS/MS sensitivity by manufacturer data, which is attributed to enhanced ion transmission and an optimized Zeno trap design. We expect that this next-generation instrument will further push the boundaries of what is achievable in high-sensitivity, high-throughput proteomics.

## Declaration of Generative AI and AI-Assisted Technologies in the Writing Process

In the preparation of this manuscript, ChatGPT 4o was utilized to assist with language refinement and editing. The authors take full responsibility for the content of the manuscript and have thoroughly reviewed and edited all outputs generated by the AI tool to ensure their accuracy, alignment with academic standards, and contextual appropriateness.

## Data Availability

The mass spectrometry proteomics data have been deposited to the ProteomeXchange Consortium via the PRIDE (33) partner repository with the dataset identifier PXD063580. (Username: reviewer_pxd063580@ebi.ac.uk Password: RKCJ2KVlPGvI) The deposited dataset encompasses the following files to ensure reproducibility: (i) the raw mass spectrometry data files. (ii) the pre-constructed spectral library in text format (HeLa + K562-1prcGlobProt-5prcLocPep-PeakViewConverted.txt) used for DIA-NN searches; (iii) the Spectronaut session file (.sne) containing all annotated spectra and processing parameters; (iv) Skyline documents (.sky) with detailed peak boundaries and integration results for all targeted assays, and (v) the Supplementary File_Targetd 184 peptides check list and supplementary peptide quantification tables referenced in the manuscript.

## Supplemental data

This article contains supplemental data.

## Conflict of interest

The authors declare the following financial interests/personal relationships which may be considered as potential competing interests: R.T. is the founder of BayOmics, Inc. The other authors declare no competing interests.

## Supplemental data

Supplemental Table 1 **The Three SWATH acquisition methods with variable window settings.** The three methods tested used 38, 65, and 85 acquisition windows, respectively. For each method, the start and end m/z of each window are provided.

Supplemental Table 2 **Protein quantification data matrix for cell-type resolved proteome**. Protein abundances were exported from Spectronaut 18 using the default quantification table format and are reported as raw label-free quantification intensities. Each row corresponds to a protein, identified by UniProt accession number. Each column corresponds to a biological replicate sample from the indicated cell type by name.

Supplemental Table 3 **Peptide quantification data matrix for cell-type resolved proteome.** Peptide-level quantification results corresponding to the protein-level data presented in Supplemental Table S2. Peptide abundances were exported from Spectronaut 18 using the default quantification table. Data type is same as Supplemental Table S2.

Supplemental Figures

Targeted MS Manuscript Checklist
