# Supplementary material for: Evaluation of the Nano Liquid Chromatography Coupled With Zeno TOF 7600 Mass Spectrometer for Cell Type-Resolved Spatial Proteomics
Source: Mol Cell Proteomics. 2026 Oct 13;25(11):101599. doi: 10.1016/j.mcpro.2026.101599 (PMC13770580; doi:10.1016/j.mcpro.2026.101599)
Supplement: Supplementary file 1 [file mmc6.pdf]

MCP MOLECULAR & CELLULAR PROTEOMICS

Targeted MS Manuscript Checklist
(Refer to Targeted MS Manuscript Guidelines for details)

Section I: General information (to be provided for all papers)

		Page	
Authors have stated the Tier level of the analyses they have developed and applied (i.e., Tier 1, Tier 2 or Tier 3 or a combination thereof).	Y	4	
Authors have supplied the names of the databases (in the manuscript) and the reviewers' login details (in the cover letter) for both the raw data and, if applicable, chromatogram files if the latter was not uploaded as supplementary material with the manuscript.	Y	Y	
If human samples are used, authors have completed the Clinical Compliance check, including IRB approval and how biofluids or tissue specimens were collected and handled. See http://www.mcponline.org/clinical for more information.	Y	Y	N/A

Section II: Chromatograms used in quantification of analytes (Tiers 1 and 2, only)

		Page	
If analyses reported are Tier 1 or Tier 2, authors have provided all chromatograms used in quantification of analytes and the names of the databases used and access codes, if applicable.	Y		N/A
If authors adjusted integration boundaries manually, was this noted and were the methods used described?	Y		N/A

Section III. Experimental design and rationale

		Page	
Authors provided a subsection in the Experimental Methods section with the header "Experimental Design and Rationale".	Y	4	
Authors described the criteria used to select the target peptides (e.g., uniqueness to proteoform, how PTM sites were avoided)	Y	Y	
The sample size (n) (for each experiment), as a number (ranges are not acceptable)	Y	Y	
The rationale for that (n) choice (e.g., statistical power of detection)	Y	Y	
Numbers and types of controls employed	Y	Y	
The number of replicates acquired, including a clear distinction between biological, process and/or technical replicates is provided	Y	Y	
Authors described if samples were processed/run in a blinded fashion or not	Y	N/A	N/A
The criteria for the inclusion or exclusion of data points (if relevant)	Y	N/A	N/A
A description of the methods used for analysis, and the choice of the statistical cutoffs (p-value, FDR etc.) for both identification and quantification (e.g., t-test) are stated.	Y	Y	

MCP MOLECULAR & CELLULAR PROTEOMICS

		Page	
Justification for the statistical methods used for analysis is provided	Y	Y	
Software used is described, including versions, and an explicit description of options and parameters used. Source code for 'in-house scripts' employed has been provided, if applicable.	Y	Y	

Section IV. Information on development and analytical validation of targeted MS assays/measurements

		Page	
Type of Targeted MS Experiment Used (i.e., PRM, MRM/SRM)	Y	Y	N/A
How the resulting precursor ions and the transitions were selected (e.g., in silico, from discovery experiments, from database/spectral library/literature) is described	Y	Y	N/A
Methods used to optimize transition ion abundance are described	Y	N/A	N/A
Method(s) used to identify interferences are described	Y	Y	
The transitions monitored for each analyte are provided (text of supplementary table)	Y	Y	
Precision of measurements was determined and reported/listed	Y	N/A	N/A
The nature of the response-to-concentration response (e.g., linearity) was determined and reported, the curve fitting algorithm used is described and the response curves (if generated) are provided	Y	N/A	N/A
Chromatography and desalting methods (on-line as well as off-line, if employed) are fully described (including packing material, column dimensions, flow rates, mobile phases, etc.)	Y	Y	N/A
Internal standards used (if any) are identified and how they were used is fully described	Y	Y	N/A
The methods to characterize the internal standards, if used, are described (e.g., amino acid analysis; MALDI or ESI MS; HPLC-UV)<br>• Note - for detailed information of characterization of internal standards, see Hoofnagle et al. Clinical Chemistry 62:1 48–69 (2016)	Y	Y	N/A
How the concentration and stability of heavy peptide standards and/or labeled proteins were assessed initially and over the course of the study is described	Y	N/A	N/A
If limits of detection and quantification were determined, authors have provided the values determined including the calculations used	Y	NA	N/A
If an evaluation of the system suitability for analysis (i.e., robustness, reproducibility, stability, carryover, etc.) was performed, authors have described the test and provided details or citation(s).<br>• For example, see Abbatiello et al. Mol Cell Proteomics. 2013 12(9): 2623–2639; and Percy et al. J Proteomics. 2013 95: 66-76	Y	N/A	N/A

MCP MOLECULAR & CELLULAR PROTEOMICS

For studies carried out over weeks or months, authors have described the samples, methods and frequency of QC of the overall system performance and quantitative reproducibility over time<br>• For example, see Simon Mol Cell Proteomics 2012	Y	N/A	N/A

Section V. Methods of data collection and data analysis

		Page	
Software used for peak picking and determination of peak area ratios is described	Y	Y	N/A
Description of how transitions were selected and used to derive quantitative information is provided (e.g., were summed, peak area ratios used; if a single transition, how was it chosen (CV, intensity, interference free, other?). Any data filtering and processing used is described.	Y	N/A	N/A
Whether a single peptide or multiple peptides were used to generate a protein concentration is reported, and how the supporting/qualifying peptides were used is described	Y	Y	N/A
If external calibration was used in the quantification, how this was done is clearly explained (including what calibrators were used, how equations were used to fit calibration curves, what QC samples were used to assess performance of the curves, what the acceptability criteria were for the QC samples, etc.)	Y	N/A	N/A
The precision (e.g., standard deviation, coefficient of variation, standard error) of the measurements for each peptide/protein is reported	Y	N/A	N/A
