# Supplementary material for: Evaluation of the Nano Liquid Chromatography Coupled With Zeno TOF 7600 Mass Spectrometer for Cell Type-Resolved Spatial Proteomics
Source: Mol Cell Proteomics. 2026 Oct 13;25(11):101599. doi: 10.1016/j.mcpro.2026.101599 (PMC13770580; doi:10.1016/j.mcpro.2026.101599)
Supplement: Supplementary file 2 [file mmc5.docx]

**Supplemental Figures**

Figure S1 zenoSWATH and ZTscan comparison by 50ng K562

Figure S2 Comparision of 10ms with100ms accumulation method on and off

Figure S3 peptide DGTFAVDGPGVIAK peak quality and intensity and peptide GAGSSEPVTGLDAK peak quality and intensity

Figure S4 mIHC staning with ZT18-2 sample

Figure S5 PG.Quantity of TGFB1 original value of spectronuant result

Figure S6 Volcano plot of CAF vs Immune groups
